# Functional Coupling between the P2X_7_ Receptor and Pannexin-1 Channel in Rat Trigeminal Ganglion Neurons

**DOI:** 10.3390/ijms22115978

**Published:** 2021-06-01

**Authors:** Hiroyuki Inoue, Hidetaka Kuroda, Wataru Ofusa, Sadao Oyama, Maki Kimura, Tatsuya Ichinohe, Yoshiyuki Shibukawa

**Affiliations:** 1 Department of Physiology, Tokyo Dental College, Tokyo 101-0061, Japan; inouehiroyuki@tdc.ac.jp (H.I.); kuroda@kdu.ac.jp (H.K.); ofusaw@tdc.ac.jp (W.O.); sadaoohyama@tdc.ac.jp (S.O.); tsumuramaki@tdc.ac.jp (M.K.); 2 Department of Dental Anesthesiology, Tokyo Dental College, Tokyo 101-0061, Japan; ichinohe@tdc.ac.jp; 3 Department of Dental Anesthesiology, Kanagawa Dental University, Kanagawa 238-8580, Japan

**Keywords:** P2X receptor, pannexin-1 channel, trigeminal ganglion neuron, patch-clamp technique, pain, tooth pain

## Abstract

The ionotropic P2X receptor, P2X_7_, is believed to regulate and/or generate nociceptive pain, and pain in several neuropathological diseases. Although there is a known relationship between P2X_7_ receptor activity and pain sensing, its detailed functional properties in trigeminal ganglion (TG) neurons remains unclear. We examined the electrophysiological and pharmacological characteristics of the P2X_7_ receptor and its functional coupling with other P2X receptors and pannexin-1 (PANX1) channels in primary cultured rat TG neurons, using whole-cell patch-clamp recordings. Application of ATP and Bz-ATP induced long-lasting biphasic inward currents that were more sensitive to extracellular Bz-ATP than ATP, indicating that the current was carried by P2X_7_ receptors. While the biphasic current densities of the first and second components were increased by Bz-ATP in a concentration dependent manner; current duration was only affected in the second component. These currents were significantly inhibited by P2X_7_ receptor antagonists, while only the second component was inhibited by P2X_1, 3,_ and _4_ receptor antagonists, PANX1 channel inhibitors, and extracellular ATPase. Taken together, our data suggests that autocrine or paracrine signaling via the P2X_7_-PANX1-P2X receptor/channel complex may play important roles in several pain sensing pathways via long-lasting neuronal activity driven by extracellular high-concentration ATP following tissue damage in the orofacial area.

## 1. Introduction

Adenosine triphosphate (ATP), which is generated intracellularly, plays important roles in both physiological and pathological cellular functions including the extracellular mediator, such as nociceptive, inflammatory, and neuropathic pain as well as migraine- and cancer-induced pain [1,2,3]. As an essential pain generator and/or modulator, extracellular ATP acts on P2 purinergic receptors, which are cell-surface nucleotide receptors [4]. P2 purinergic receptors can be classified into P2X and P2Y receptors, which are further divided into seven subtypes (P2X_1_, P2X_2_, P2X_3_, P2X_4_, P2X_5_, P2X_6_, and P2X_7_) of ligand-gated cation channels and eight subtypes (P2Y_1_, P2Y_2_, P2Y_4_, P2Y_6_, P2Y_11_, P2Y_12_, P2Y_13_, and P2Y_14_) of G-protein-coupled receptors [4]. Among the P2 purinergic receptors, P2X_3′_s involvement in nociceptive pain was first reported in the tooth-pulp afferents of trigeminal ganglion (TG) neurons [1]. While protein and/or mRNA expression of P2X_1_–_6_, and P2Y_12_ receptors has been reported in TG neurons [5,6,7,8,9,10], the detailed functional electrophysiological and pharmacological properties of P2X_7_ receptors in TG neurons has yet to be examined.

The P2X_7_ receptor, initially identified as the P2Z receptor, is a trimeric ligand-gated cation channel expressed on the cell membranes of various cells [11,12,13]. The P2X_7_ receptor can be distinguished from other P2X receptor subtypes as follows: (1) requires high concentrations of extracellular ATP to be activated, (2) shows high sensitivity to 2(3)-O-(4-Benzoylbenzoyl) adenosine 5-triphosphate triethylammonium salt (Bz-ATP), and (3) forms channel pores that allow the permeation of cations and larger molecules, such as ATP and glutamate [11,14,15,16,17,18,19]. Importantly, the P2X_7_ receptor has been shown to modulate trigeminal nociceptive processing [20]. Interestingly, expression of the P2X_7_ receptor is greater in satellite glial cells of the TG than that in neuronal cells [21,22]. Furthermore, extracellular ATP can activate P2X_7_ receptors in the surrounding glia under migraine-like conditions. Therefore, activation of the glial P2X_7_ receptor is thought to play an important role in physiological processes and numerous diseases, including inflammatory and neurological diseases as well as neuropathic pain in the orofacial area [9,23,24,25,26]. Although the characteristics of the P2X_7_ receptor have been well demonstrated in glial cells of the TG, the role of the P2X_7_ receptor in TG neurons during nociceptive and neuropathic pain as well as its detailed biophysical, pharmacological, and functional properties, remains to be clarified.

The receptor-receptor (or receptor-channel) interactions of the P2X_7_ receptor are also controversial, including its interactions with the pannexin-1 (PANX1) channel and other P2X receptor(s) [9,25,27,28,29,30]. The PANX1 channel, a transmembrane channel allowing the passage of ions and small molecules like ATP, is involved in immune responses through an interaction with the P2X_7_ receptor [9,25,29,30,31]. The P2X_4_ receptor is also involved in cell death and inflammation via coupling with the P2X_7_ receptor [2,27,28,32,33]. However, the biophysical characteristics and interactions between P2X receptors and PANX1 channels in trigeminal nociceptive processing remains unclear. 

Therefore, this study was designed to examine the electrophysiological and pharmacological characteristics of the P2X_7_ receptor and its functional coupling with the PANX1 channel as well as other P2X receptors in TG neurons.

## 2. Results

### 2.1. Cell Identification of TG Neurons

**Figure 1:**
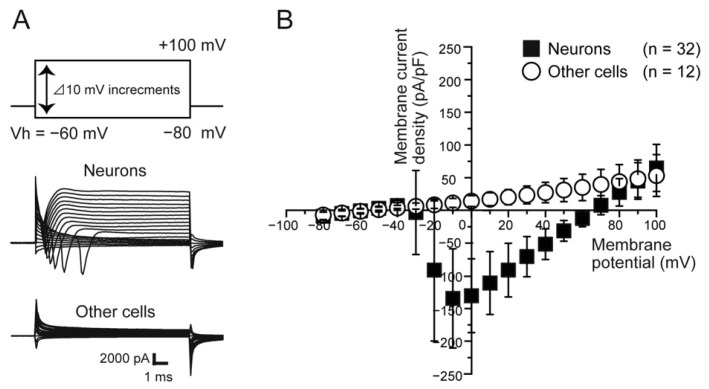
Characterization of primary cultured TG neurons. (**A**) Traces illustrate whole-cell currents evoked by a sequence of 10-ms depolarizing voltage pulses at Vh of −60 mV in 10 mV increments from −80 to +100 mV. The upper traces show the voltage pulse protocol. The middle traces were obtained from the cell in which the voltage-dependent inward currents were generated, and the lower traces were the cells in which the inward currents were not observed. Cells in which the generated inward current was observed were identified as neurons in the TG cells. (**B**) Current-voltage (I–V) relationships were obtained by plotting the values of the peak current densities against the applied membrane potentials. Cell size was determined by normalizing the measured capacitance and expressing current amplitudes in terms of current densities (pA/pF). At a Vh of −60 mV, black squares show an inward current developed at a membrane potential of −30 mV and reached maximum densities at −10 mV, leading to the identification of TG neurons. White circles show the I–V relationships with cells showing no inward current. Each data point represents the mean ± S.D. of the 32 experiments for the cells showing inward current and 12 experiments for cells not showing it.

TG neurons were identified as cells that expressed voltage-dependent ionic currents. The voltage-dependent inward currents (I_inward_) were recorded at a holding potential (Vh) of −60 mV, and neurons were identified as cells showing inward currents (Figure 1A). The current–voltage (I-V) relationships, which were determined by plotting the peak current amplitudes in the densities against the applied membrane potentials, were measured by applying 20-ms voltage steps ranging from −80 to +100 mV at 2-s intervals (Figure 1A,B). At a Vh of −60 mV, the inward currents in the neurons developed at a membrane potential of −30 mV and reached maximum amplitude at a membrane potential of −10 mV (Figure 1B). Cells not showing inward currents were not taken into account in further analyses.

### 2.2. Response of TG Neurons during Application of ATP and Bz-ATP

**Figure 2:**
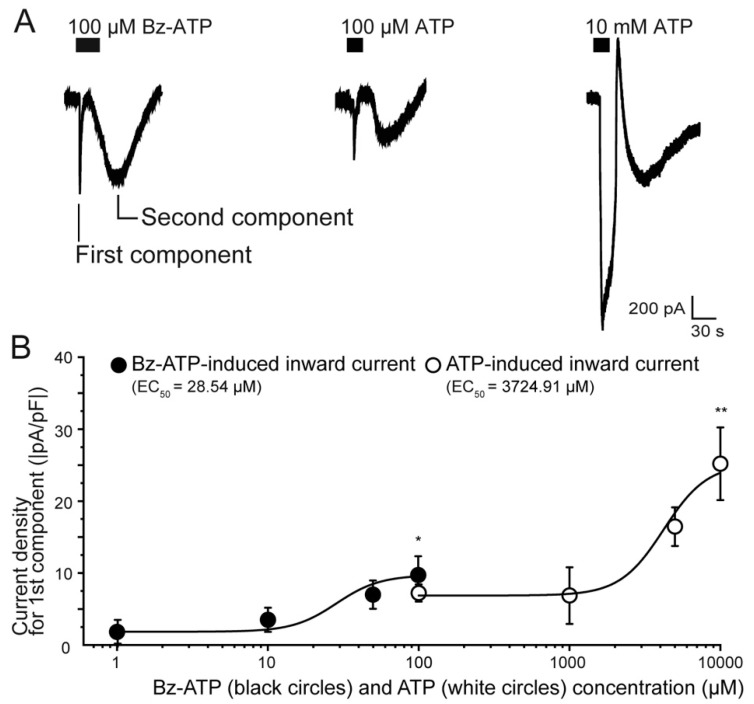
ATP- and Bz-ATP-induced currents. (**A**) Representative traces of inward currents induced by 100 µM Bz-ATP, 100 µM ATP, and 10 mM ATP. Applied ATP and Bz-ATP protocols are shown in the upper black boxes; (**B**) Dose-response relationships between the peak current densities of the first current component of the agonist-induced inward currents vs. each concentration of Bz-ATP (1, 10, 50, and 100 µM) and ATP (100 µM, 1 mM, 5 mM, and 10 mM). Curves (solid lines) on the semilogarithmic scale were fitted to the equation described in the text. Note that a high concentration of ATP (10 mM) elicited inward currents, which showed slow inactivation and long-lasting properties. Each data point represents the mean ± SD of five experiments. Statistically significant differences between data points are indicated by asterisks. * *p* < 0.05 vs. 1 µM Bz-ATP; ** *p* < 0.05 vs. 100 µM ATP.

We examined the concentration-dependent effects of both ATP and Bz-ATP on the induced-current response in TG neurons. In the standard ECS, application of 100 µM Bz-ATP (a highly potent P2X_7_ receptor agonist), as well as 100 µM or 10 mM, of ATP evoked biphasic inward currents at Vh of −60 mV (Figure 2A), composing the transient first component and the persistent second component, respectively. The currents were elicited by four different concentrations of Bz-ATP (1–100 µM) and ATP (100 µM–10 mM). The amplitudes of current densities increased with increasing Bz-ATP and ATP concentrations in a concentration-dependent manner (Figure 2B). The EC_50_ of ATP on its induced-currents was 3.72 mM, whereas the EC_50_ of Bz-ATP on its induced-currents was 28.5 µM. This is a 160-fold difference in the EC_50_ values of Bz-ATP and ATP. 

### 2.3. Concentration-Dependent Effect of Bz-ATP on the Biphasic Inward Current

**Figure 3:**
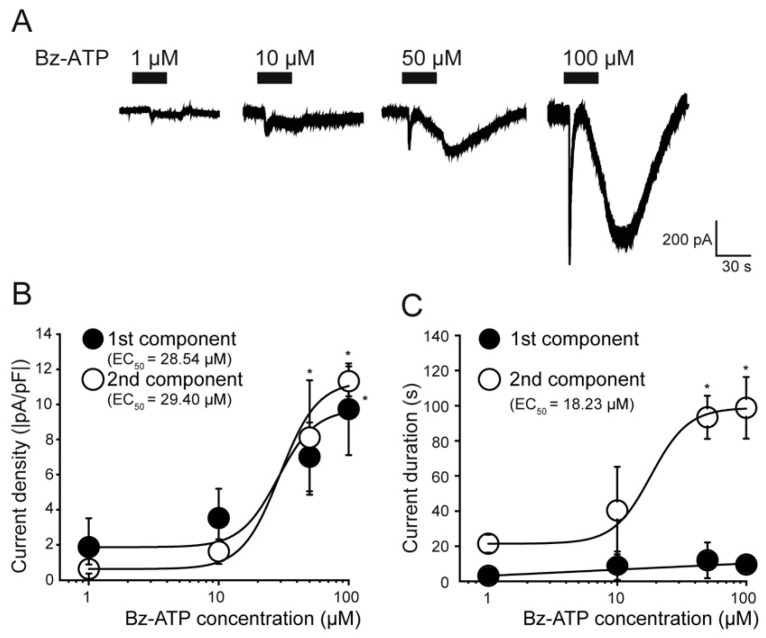
Dose-dependent relationships for current densities and duration of first and second components of the Bz-ATP-induced inward current. (**A**) Representative traces of inward currents induced by the application of four different concentrations of Bz-ATP (1–100 µM) are shown. The applied Bz-ATP protocols are shown in the upper black boxes. (**B**,**C**) Showing the concentration-response relationships of applied extracellular Bz-ATP. Data points illustrate current densities (**B**) and durations (**C**) as functions of the applied concentration of Bz-ATP. Black circles represent the first component; white open circles indicate the second component of the biphasic inward current. Curves (solid lines) on the semilogarithmic scale were fitted according to the equation described in the text, except for the duration of the second component (which was fitted to a linear regression). Note that the current densities for the first component of the Bz-ATP-induced current in B (black circles) are identical to the data shown in Figure 2B. Each data point represents the mean ± SD of five separate experiments. Statistically significant differences between data points are indicated by asterisks. * *p* < 0.05.

To investigate the detailed concentration-dependent effect on the current response, we examined four different concentrations of Bz-ATP (1–100 µM). Biphasic inward currents were elicited by different concentrations of Bz-ATP (Figure 3A). The current densities in both the first and second components of the Bz-ATP-induced inward currents increased in a concentration-dependent manner (Figure 3B). The EC_50_ obtained in the first and second components were 28.5 µM and 29.4 µM (Figure 3B), respectively. Additionally, the duration of the second component increased in a concentration-dependent manner (EC_50_ of 18.2 µM of Bz-ATP), while that of the first component remained consistent (EC_50_ was not calculated; Figure 3C).

### 2.4. Effects of P2X Receptor Antagonists on the Bz-ATP-Induced Currents

**Figure 4:**
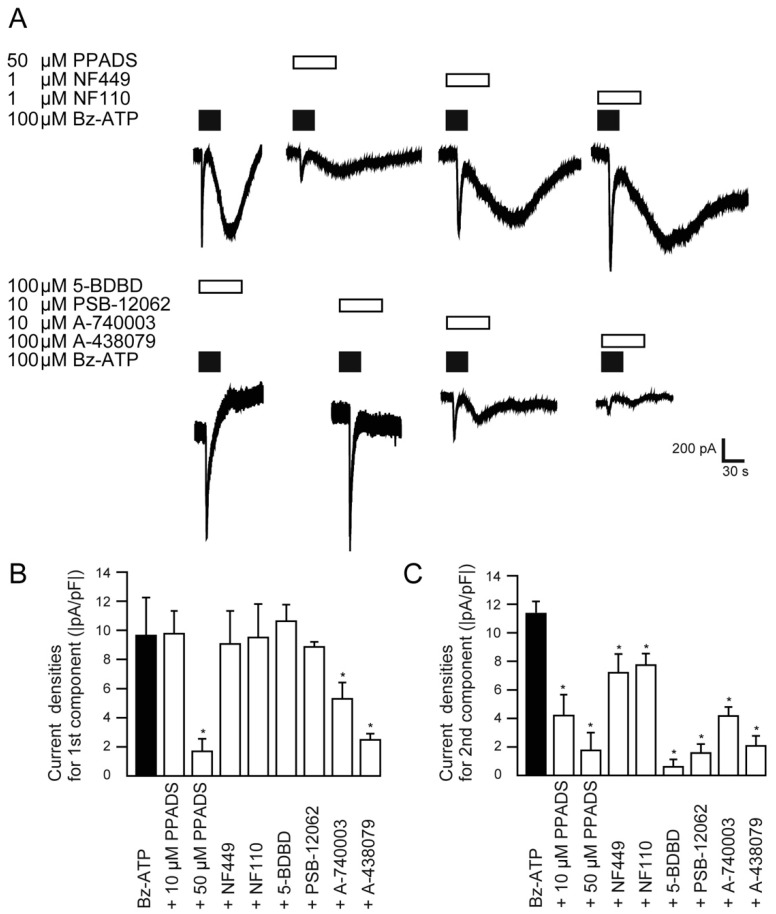
Pharmacological identification of the first and second component of Bz-ATP-induced currents. (**A**) Representative traces of Bz-ATP-induced currents (upper black boxes; 100 μM) with or without non-specific P2X receptor antagonists, P2X_1_-, -P2X_3_-, P2X_4_- and P2X_7_-receptor antagonists (upper open boxes: 50 µM PPADS, 1 µM NF449, 1 µM NF100, 100 µM 5-BDBD, 10 µM PSB-12062, 10 µM A-740003, or 100 µM A-438079). (**B**,**C**) Summary bar graphs show current densities of the first (**B**) and second components (**C**) of the Bz-ATP-induced biphasic inward currents with or without P2X receptor antagonists (see above). Each bar denotes the mean ± SD of five experiments. Statistically significant differences between columns are indicated by asterisks. * *p* < 0.05.

To investigate which P2X receptors contributed to the biphasic inward current induced by Bz-ATP, we examined the effects of the non-specific P2X receptor antagonist (PPADS), the P2X_1_ receptor antagonist (NF449), the P2X_3_ receptor antagonist (NF110), the P2X_4_ receptor antagonists (5-BDBD and PSB-12062, respectively), and P2X_7_ receptor antagonists (A-740003 and A-438079, respectively). In the first component, the inward currents induced by 100 µM Bz-ATP were significantly inhibited by 50 µM PPADS, 10 µM A-740003, and 100 µM A-438079 (Figure 4A,B). The second component was significantly inhibited by 10 µM and 50 µM PPADS, 100 µM 5-BDBD, 10 µM PSB-12062, 10 µM A-740003, and 100 µM A-438079, while a slight but significant inhibition was observed with the application of 1µM NF449 and 1 µM NF 100 (Figure 4A,C).

### 2.5. P2X_7_-PANX1-P2X Receptor/Channel Axis Mediates Biphasic Inward Current Activation as Autocrine Signaling

**Figure 5:**
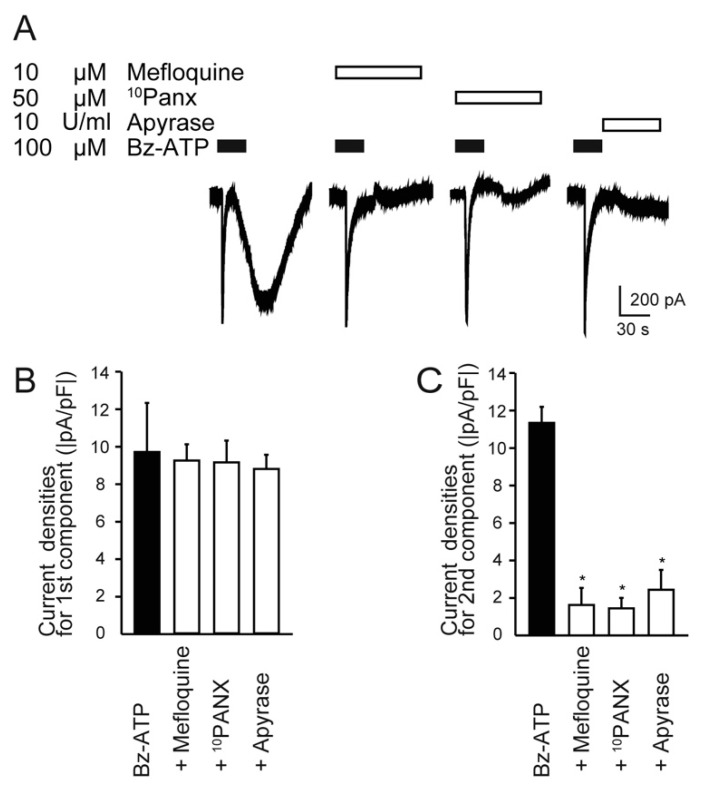
Pharmacological properties of Bz-ATP-induced biphasic inward current. (**A**) Representative traces of Bz-ATP-induced current (upper black boxes; 100 μM) with or without PANX1 channel inhibitors (10 μM mefloquine and 50 μM ^10^ Panx), and ATP-degrading enzyme (10 U/mL apyrase) (each upper open box). (**B**,**C**) Summary bar graphs showing the current densities of the first (**B**) and second components (**C**) of the Bz-ATP-induced biphasic inward currents. Each bar denotes the mean ± SD of five experiments. Statistically significant differences between columns are indicated by asterisks. * *p* < 0.05.

To investigate the nature of the second component in the biphasic inward current, we examined the effect of extracellular ATP degradation enzymes and PANX1 antagonists on the current induced by Bz-ATP (Figure 5A). Although there was no significant effect on the first component of the biphasic inward currents induced by 100 µM Bz-ATP (Figure 5B), the current densities of the second components were significantly reduced by 10 µM mefloquine (non-specific PANX1 antagonist) and 50 µM ^10^ Panx (specific PANX1 antagonist), as well as 100 U/mL apyrases (extracellular ATP degradation enzyme) (Figure 5C). 

## 3. Discussion

This study revealed the functional expression and synergistic effects of P2X_7_, PANX1 channels, and other P2X receptors in rat TG neurons. We found that the P2X_7_-PANX1-P2X receptor/channel axis mediates the Bz-ATP-induced long-lasting biphasic inward current response in an autocrine manner. 

P2X_7_ receptors are activated by extracellular ATP (at a high concentration) and Bz-ATP, and are found in numerous organs [2,16,17,25,31,34], with the exception of TG neurons [22]. P2X_7_ receptors require activation at ATP concentrations greater than 100 μM, while Bz-ATP is approximately 10–30 times more potent than ATP [2]. In line with these observations, we found that the EC_50_ required to activate an inward current in TG neurons was 20 μM for Bz-ATP, but 3300 μM for ATP (Figure 2). In addition, the first component of the biphasic inward currents induced by 100 μM Bz-ATP was reduced by P2X_7_ receptor antagonists, A-438079 and A-740003 (Figure 4). These results indicate that P2X_7_ receptors in TG neurons are likely less sensitive to extracellular ATP but are highly sensitive to extracellular Bz-ATP. Furthermore, Bz-ATP also triggers the activation of biphasic inward currents. 

We also found that application of Bz-ATP and high concentrations of ATP to TG neurons elicited an additional current component, that is, the second component, of the biphasic inward currents in a concentration-dependent manner (Figure 3). Importantly, the second component of biphasic inward currents induced by Bz-ATP was affected by other P2X receptor antagonists (predominantly the P2X_4_ antagonist, but also the P2X_1_ and P2X_3_ antagonists) (Figure 4) as well as the PANX1 channel inhibitor and extracellular ATPase (Figure 5), although the first component did not show any sensitivity to these antagonists (Figure 4). It has been reported that P2X receptor-induced activation of the PANX1 channel is a relatively late event, which is preceded by the rapid opening of the ionotropic receptor, and occurs only after prolonged agonist application [35]. The PANX1 channel assists with autocrine and/or paracrine signaling by providing an extracellular signaling pathway via ATP [36,37,38]. Moreover, it has been reported that functional interactions between the P2X_7_ and/or P2X_4_ receptor and PANX1 channel form a complex that interact directly with each other [30,39]. Together with these reports, our results suggest that activation of the P2X_7_ receptor most likely enhances PANX1 channel opening enabling the extracellular release of ATP which activates other P2X receptors, especially the P2X_4_ receptor, resulting in the generation of biphasic inward currents through an autocrine mechanism.

The P2X_7_-PANX1-P2X receptor/channel has important roles in both physiological and pathological conditions [35,40,41]. In the inflammatory responses, the interaction between P2X_7_ receptors and PANX1 channels promotes cytosolic multiprotein oligomerization that leads to the activation of the inflammasome, followed by the extracellular release of cytokines [42]. In contrast, besides inducing inflammasome, ATP also influences the activation and chemoattraction of leukocytes to the injury site to amplify the immune response. Multinucleated macrophage formation takes place following ATP release through P2X_7_ receptors and subsequent conversion into adenosine to enhance the expression of CD44, while PANX1 allows the permeabilization of the plasma membrane, thereby enabling fusion [42]. These inflammatory- and/or immuno-responses lead to inflammation-induced cell death, that is pyroptosis. In pyroptosis, released ATP via PANX1 channels activates P2X receptors, which underlies the formation of a membrane pore that triggers cytolysis [42]. It has also been reported that the activation of the neuronal P2X_7_-PANX1 axis mediates the death of enteric neurons during colitis [25], while the activation of the P2X_7_-PANX1 complex enhances spreading depolarization and neuroinflammation [9] of the trigeminovascular system, leading to migraine and stroke. Therefore, there is considerable interest in understanding whether the mechanisms of pyroptosis in the peripheral trigeminal nervous system are involved in the generation and/or modulation of neuropathic pain (represented as postherpetic neuralgia, trigeminal neuralgia, and postoperative neuropathic pain) and/or trigeminal autonomic cephalalgias. This study was conducted to determine the electrophysiological and pharmacological characteristics of the P2X_7_ receptor and its functional coupling mediated by the P2X_7_-PANX1-P2X receptor/channel complex in trigeminal ganglion neurons. Thus, to reveal the detailed role of the P2X_7_-PANX1-P2X axis in the generation of pain under both physiological and pathophysiological conditions, further studies, such as analyzing pain behaviors induced by stimuli in the orofacial area (personal communication from YS), are of immediate interest.

This study has several limitations. First, the first component of the Bz-ATP-induced biphasic current was not completely suppressed by the P2X_7_ receptor-selective antagonists, A-438079 and A-740003. Therefore, a residual current component could still be observed (Figure 4), suggesting that the first component of the current may not be completely mediated by the P2X_7_ receptor. In fact, Bz-ATP has been reported to act on various recombinant homomeric P2X receptors (i.e., P2X_1_, P2X_2_, P2X_3_, P2X_4_, P2X_5_, and P2X_7_), and the presence of heteromeric P2X_4/7_ receptors has been proposed [43]. Furthermore, P2X_1_–P2X_6_ receptor subtypes have been reported in rat TG neurons [1,7,8]. Moreover, large pore openings giving a biphasic current can also be generated by P2X_2_ and P2X_4_ receptors [44]. Finally, P2X_2_ receptors are also expressed in TG neurons [45]. In this study, however, the first component of the Bz-ATP-induced current did not show sensitivity to P2X_1_, P2X_3_, and P2X_4_ receptor-selective antagonists. PPADS, a non-selective P2X receptor antagonist, blocks recombinant P2X_1_, P2X_2_, P2X_3_, and P2X_5_ receptors (IC_50_ = 1–3 μM), and is effective against P2X_7_ at high concentrations (IC_50_ = 50 μM) [43]. Based on the present results showing that the first component could not be inhibited by low concentrations (10 µM) of PPADS but was inhibited by high concentrations (50 µM), the contribution of P2X_2_ and P2X_5_ receptor activation to the first component of the Bz-ATP-induced inward current is unlikely. Thus, we suggest that the activation of the first component is predominantly P2X_7_ receptor-dependent, and further studies are needed to clarify the contribution of P2X_2_ and P2X_5_ receptors to the first component of the Bz-ATP-induced biphasic current. Since selective antagonists for P2X_2_ and P2X_5_ receptors are not commercially available, detailed contributions of these P2X subtypes could not be pursued in this study. The concentration of P2X_7_ receptor antagonists used in this study was 10–100 µM, while the reported IC_50_ for these reagents is approximately 50 nM [43]. This is because the currents were not sufficiently inhibited until the concentration was raised up to the µM order in rat TG neurons. Importantly, the IC_50_ of some P2X receptor antagonists has also been reported to be highly species-dependent. The amino acid residue at position 95 of the P2X_7_ receptor is key and determines species-dependent selectivity of allosteric antagonists (such as GW791343, KN62, and SB203580). The P2X_7_ receptor antagonists, A740003 and A438079, which we used in this study, also show allosteric effects [46]. Therefore, the species-dependent selectivity of the allosteric antagonists might be the reason as to why the 10–100 µM order of P2X_7_ receptor antagonist concentration was necessary to inhibit the Bz-ATP-induced biphasic currents in this study. Examples using 30 µM A438079 to inhibit P2X_7_ receptor activity have also been recently reported [25]. In addition, in line with our present results (see above too), this residue (at position 95) does not affect the species-dependent selectivity of PPADS, which is a slowly reversible antagonist of the P2X_7_ receptor and acts at the ATP-binding site [47].

Second, we showed that extracellular ATP released from the PANX1 channel can activate the P2X_1_, P2X_3_, and P2X_4_ receptors in the same TG neuron as an autocrine signal that is triggered by P2X_7_ receptor activation. However, we were unable to estimate the amount and extracellular concentration of ATP released from the PANX1 channel, as well as its diffusible constant. The basal level of extracellular ATP is low, in nanomolar levels, in the trigeminal ganglion neurons to avoid constant nociceptive firing in trigeminal nerves [26]. In contrast, activation of P2 receptors usually occurs at nucleotide concentrations within the range of 10^−6^–10^−3^ M, which exceeds the nanomolar ATP level at rest ([48] for review). A recent innovative ATP-sensing assay revealed that 100–200 µM ATP is released in response to P2X_7_ receptor activation by Bz-ATP [49]. Although further studies are needed to estimate the amount of ATP released following the activation of the P2X_7_-PANX1 axis, up to µM order of ATP release might be reasonable to activate P2 receptors expressed in TG neurons.

In conclusion, our study suggests that the functional P2X_7_-PANX1-P2X complex elicits long-lasting biphasic inward currents in TG neurons. This autocrine and/or paracrine signaling via ATP may contribute to the pain mechanism in the orofacial region.

## 4. Materials and Methods

### 4.1. Th Cell Isolation and Primary Culture

All animals were treated in accordance with the Guiding Principles for the Care and Use of Animals in the Field of Physiological Sciences approved by the Council of the Physiological Society of Japan and by the American Physiological Society. This study also followed the guidelines established by the National Institutes of Health (Bethesda, MD, USA) regarding the care and use of animals for experimental procedures. The study was approved by the Ethics Committee of Tokyo Dental College (approval No.282804, 292502, 302502, 192701). 

TG cells were isolated from neonatal Wistar rats (7 days old) under pentobarbital sodium anesthesia (50 mg/kg intraperitoneally) following the administration of isoflurane (3.0% vol). TG cells were dissociated using enzymatic treatment with Hank’s balanced salt solution (HBSS) (137 mM NaCl, 5.0 mM KCl, 2.0 mM CaCl_2_, 0.5 mM MgCl_2_, 0.44 mM KH_2_PO_4_, 0.34 mM Na_2_HPO_4_, 4.17 mM NaHCO_3_, 5.55 mM glucose [pH was adjusted to 7.4 using tris]) containing 20 U/mL papain (Worthington, Lakewood, NJ, USA) for 20 min at 37 °C, followed by dissociation by trituration [7,10,50,51,52]. Primary cultures of the TG cells were performed using Leibovitz’s L-15 medium (Thermo Fisher Scientific, Carlsbad, CA, USA), containing 10% fetal bovine serum (Thermo Fisher Scientific), 1% amphotericin B (Sigma-Aldrich Co., St Louis, MI, USA), 1% penicillin-streptomycin (Thermo Fisher Scientific), 24 mM NaHCO_3_, and 30 mM glucose (pH 7.4). The cells were incubated and maintained for 48 h at 37 °C in a humidified atmosphere containing 95% air and 5% CO_2_. 

### 4.2. Whole-Cell Recording Techniques

Patch-clamp recordings of the whole-cell configuration were performed under voltage-clamp conditions. Patch pipettes with a resistance of 2–5 MΩ were pulled from capillary tubes using a DMZ-Universal Puller (Zeitz Instruments, Martinsried, Germany), and the pipettes were filled with an intracellular solution (ICS; solution composition is described below). Whole-cell currents were measured using a patch-clamp amplifier (L/M-EPC-7+; Heka Electronik, Lambrecht, Germany). Current traces were displayed and stored in a computer using pCLAMP (Molecular Devices, LLC., San Jose, CA, USA) after digitizing the analog signals at 10 kHz (DigiData 1440A; Molecular Device). The current records were filtered at 3 kHz. Data were analyzed offline using pCLAMP. All experiments were performed at 25 °C.

### 4.3. Solutions and Reagents

HBSS was used as the standard extracellular solution (ECS) for patch-clamp recordings. The intracellular solution (ICS) for patch-clamp recordings contained the following: 140 mM KCl, 10 mM NaCl, and 10 mM HEPES (pH was adjusted to 7.2, using Tris). The following pharmacological agents were used: ATP disodium salt (Sigma-Aldrich Co.) and Bz-ATP (Sigma-Aldrich Co.) as P2X receptor agonist, 3-(5-(2,3-dichlorophenyl)-1H-tetrazol-1-yl) methyl pyridine hydrochloride hydrate (A-438079) (Sigma-Aldrich Co.) and N-(1-{[(cyanoimino) (5-quinolinylamino) methyl] amino}-2, 2-dimethylpropyl)-2-(3, 4-dimethoxyphenyl) acetamide (A-740003) (Sigma-Aldrich Co.) as P2X_7_ receptor antagonists, 5-(3-bromophenyl)-1,3-dihydro-2*H*-benzofuro[3,2-*e*]-1,4-diazepin-2-one (5-BDBD) (Tocris Bioscience, Bristol, UK) and 10-[(4-Methylphenyl)sulfonyl]-10H-phenoxazine (PSB-12062) (Sigma-Aldrich Co.) as P2X_4_ receptor antagonists, 4-[[4-Formyl-5-hydroxy-6-methyl-3-[(phosphonooxy)methyl]-2-pyridinyl]azo]-1,3-benzenedisulfonic acid tetrasodium salt, PPADS tetrasodium salt hydrate (PPADS) (Sigma-Aldrich Co.) as P2X receptor antagonist, 4,4′,4″,4‴-[Carbonylbis(imino-5,1,3-benzenetriyl-*bis*(carbonylimino))]*tetrakis*-1,3-benzenedisulfonic acid, octasodium salt (NF449) (Tocris Bioscience, Bristol, UK) as P2X_1_ receptor antagonist, 4,4′,4″,4‴-[Carbonyl*bis*[imino-5,1,3-benzenetriyl*bis*(carbonylimino)]]tetrakisbenzenesulfonic acid tetrasodium salt (NF110) (Tocris Bioscience, Bristol, UK) as P2X_3_ receptor antagonist, (AS)-rel-a-(2R)-2-Piperidinyl-2,8-bis(trifluoromethyl)-4-quinolinemethanol monohydrochloride (mefloquine hydrochloride) (Sigma-Aldrich Co.) and Trp-Arg-Gln-Aln-Phe-Val-Asp-Ser-Tyr (^10^ Panx) (Tocris Bioscience, Bristol, UK) as PANX1 channel inhibitors, as well as ATP diphosphohydrolase adenosine 5′-triphosphatase (apyrase) (Sigma-Aldrich Co.). These reagents were diluted in ECSs for patch-clamp recording and applied at the appropriate concentration to primary cultured TG neurons via a rapid solution exchange system (Warner Instruments, Hamden, CT, USA).

### 4.4. Analyses

Differences in cell size were accounted for by normalizing the measured capacitance. In this study, the induced current amplitudes are expressed in terms of the current densities (pA/pF). Data are expressed as the mean ± standard deviation (S.D.) of n observations, where n represents the number of separate experiments. The concentration dependence of Bz-ATP activity was obtained by fitting the data with the following function:

A = A_max_/[1 + (EC_50_/[X]_o_^h^)] + A_min_,

where EC_50_ is the half-maximal effective concentration of applied Bz-ATP, A_max_ is the maximal response, and A_min_ is the minimal response. [X]_o_ indicates the concentration of extracellular Bz-ATP.

One-way ANOVA with Bonferroni correction was used to determine parametric statistical significance; the Kruskal-Wallis test with Dunn’s posthoc test was used to determine nonparametric statistical significance. All statistical analyses were performed using GraphPad Prism version 7 statistical software package (GraphPad Software, San Diego, CA, USA).

## Data Availability

The data presented in this study are available on request from the corresponding author.
